# Optimization of deep learning methods for visualization of tumor heterogeneity and brain tumor grading through digital pathology

**DOI:** 10.1093/noajnl/vdaa110

**Published:** 2020-08-29

**Authors:** An Hoai Truong, Viktoriia Sharmanska, Clara Limbӓck-Stanic, Matthew Grech-Sollars

**Affiliations:** 1 Department of Surgery and Cancer, Faculty of Medicine, Imperial College London, London, UK; 2 Department of Computing, Faculty of Engineering, Imperial College London, London, UK; 3 Department of Brain Sciences, Imperial College London, London, UK; 4 Department of Histopathology, Imperial College Healthcare NHS Trust, London, UK; 5 Department of Imaging, Imperial College Healthcare NHS Trust, London, UK

**Keywords:** brain tumor, deep learning, digital pathology, machine learning, tumor heterogeneity

## Abstract

**Background:**

Variations in prognosis and treatment options for gliomas are dependent on tumor grading. When tissue is available for analysis, grade is established based on histological criteria. However, histopathological diagnosis is not always reliable or straight-forward due to tumor heterogeneity, sampling error, and subjectivity, and hence there is great interobserver variability in readings.

**Methods:**

We trained convolutional neural network models to classify digital whole-slide histopathology images from The Cancer Genome Atlas. We tested a number of optimization parameters.

**Results:**

Data augmentation did not improve model training, while a smaller batch size helped to prevent overfitting and led to improved model performance. There was no significant difference in performance between a modular 2-class model and a single 3-class model system. The best models trained achieved a mean accuracy of 73% in classifying glioblastoma from other grades and 53% between WHO grade II and III gliomas. A visualization method was developed to convey the model output in a clinically relevant manner by overlaying color-coded predictions over the original whole-slide image.

**Conclusions:**

Our developed visualization method reflects the clinical decision-making process by highlighting the intratumor heterogeneity and may be used in a clinical setting to aid diagnosis. Explainable artificial intelligence techniques may allow further evaluation of the model and underline areas for improvements such as biases. Due to intratumor heterogeneity, data annotation for training was imprecise, and hence performance was lower than expected. The models may be further improved by employing advanced data augmentation strategies and using more precise semiautomatic or manually labeled training data.

**Key Points**

- Data augmentation did not improve training, smaller batch size improved model performance.
- No significant difference in performance between 2- and 3-class models.
- We present an output visualization method that may be used clinically to aid histopathologists.

**Importance of the Study**

Interobserver variability in histopathology brain tumor grading requires an objective approach. While machine learning methods have been explored in grading brain tumors, we developed a methodology to highlight regions of the tumor which are considered more aggressive by the machine learning network. This approach highlights how clinicians make their diagnosis from digital pathology and is aimed at aiding the histopathologist to identify the regions considered to be most aggressive. The methodology also follows a discussion with a patient and public involvement group that suggested that artificial intelligence should be used to help clinicians in making their diagnosis and that they would be concerned around the use of a black-box technique to diagnose patients.

Diffuse glioma prognosis is associated with age, tumor type, WHO grade, extent of resection, and genetic alterations.^1^ Following resection and biopsy, where possible, diagnosis is given based on histopathology and signature molecular genetic alterations. The grades of tumors are currently classified according to the 2016 World Health Organization Classification of Tumors of the Central Nervous System, comprising of a 4-tiered system with grade IV, also known as glioblastoma (GBM), being the most malignant.^2^

However, the grading of gliomas can pose a challenge in clinical practice. Tumors are often heterogenous and can have characteristics of both low- and high-grade lesions in different tissue areas, making a distinction between grades difficult, especially with imprecise diagnosis criteria, particularly between WHO grades II and III.^3^ There is also an element of subjectivity and thus there are variations in grades given between different histopathologists.^4^ Effective, accurate, and objective grading of gliomas is of high importance as this determines therapeutic strategies and availability of clinical trials to the individual patients, affecting prognosis. Therefore, the development of an objective, quantitative tool to aid clinicians in the classification process is required to improve the accuracy and reliability of glioma diagnosis and we here explore deep learning methods. 

Machine learning is a statistical and computational technique to analyze and model data without prior knowledge, thus relying on the inference of patterns in the dataset.^5^ Deep learning is a subset of machine learning that involves the extraction of abstract pattern representations at multiple levels and layers, with each layer comprising of representation at a higher and more abstract level.^6^ In classification tasks of image data, for example, tumor grading using histopathology images, the hierarchical approach allows extraction of various important features (eg, edges, colors, orientation, and location) for discrimination and to suppress irrelevant, artefactual information.

There are various architectures relating to the deep learning methodology, encompassing both unsupervised and supervised strategies.^6^ Convolutional neural networks (CNNs) in particular have been demonstrated to be very effective for image recognition, classification, and computer vision.^7^ They utilize a convolution operation across multiple layers to extract different features from the input images, which are learned automatically, and the outputs are classes or categories such as tumor/nontumor, or tumor grades. The performance between CNNs and other machine learning techniques for medical imaging has been compared in various instances.^8^ As such CNNs for medical imaging are starting to gain popularity among deep learning techniques for its winning error rate^9^ and good accuracy across multiple types of medical images.^8^

The aim of this study was to develop a computational pipeline for the classification of gliomas through histopathological images and CNNs. The effect of data augmentation, hyperparameter tuning, and multiclass classification strategies on CNN model performance was investigated. Furthermore, we devised a novel method for visualizing output to tackle the inherent heterogeneity that exists within these brain tumors and to improve the explainability of the model.

## Materials and Methods

### Data Source

Hematoxylin and eosin (H&E) stained whole-slide histopathology images (WSIs) were obtained from The Cancer Genome Atlas (TCGA).^10^ Data included 785 WSIs from 249 WHO grade II patients, 773 images from 264 WHO grade III patients, and 2053 WSIs from 607 GBM patients. In differentiating between GBM and non-GBM tumors, the data from the WHO grade II and III patients were combined to include 1558 WSIs from 513 patients. In TCGA, the non-GBM samples are classified as astrocytoma, oligoastrocytoma, or oligodendroglioma. The samples are stratified according to these tumor types to give an equal distribution of each type in each training, validation, and evaluation dataset (Table 1).

**Table 1.** Number of Cases in Each Tumor Subtype and Grade, Stratified Across Training, Validation, and evaluation datasets

Grade	Type	Number of Cases			
		Total	Training	Validation	Evaluation
GII	Astrocytoma	63	45	11	6
	Oligoastrocytoma	74	53	13	7
	Oligodendroglioma	112	81	20	11
	All	249	179	45	25
GIII	Astrocytoma	131	94	24	13
	Oligoastrocytoma	55	40	10	6
	Oligodendroglioma	78	56	14	8
	All	264	190	48	26
GBM	—	607	109	61	437

Tumor types as defined on the TCGA database.

### Preprocessing

**Figure 1.**
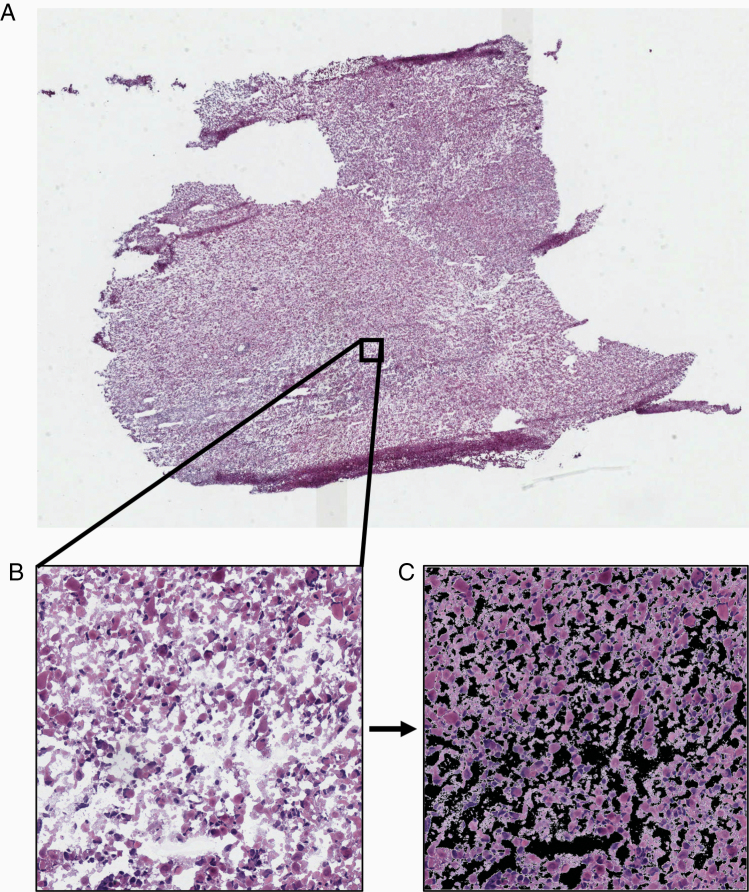
Examples demonstrating the data preprocessing. (A) Example of a whole-slide histopathology image (WSI) of a brain tumor sample imaged at 40× magnification. (B) A tile sized 1024 × 1024 pixels is extracted from the WSI at 20× magnification. (C) The background is removed in order to calculate the percentage of tissue present on the tile. Tiles with tissue percentage more than 90% were included in model development or evaluation.

Image files were in *.svs* format and had magnifications of 20× or 40× with varying dimensions (Figure 1A). In order to normalize images to appropriate dimensions and magnification for use as inputs, the WSIs were divided into tiles (Figure 1B). A tile size of 1024 × 1024 pixels, obtained at 20× magnification, was used. The background of each tile was removed, and the percentage of tissue present was calculated (Figure 1C). Tiles with 90% or more tissue present were included in model development or evaluation. A total of over 680 000 tiles across all classes were used.

### Deep Convolutional Residual Neural Networks

CNNs are regularized multilayer perceptrons, that is, networks where each neuron in a layer is connected to all neurons in the successive layer, regularized by building on the hierarchical structure to identify more complex patterns based on simpler patterns.^6^ CNNs are composed of convolutional, pooling, and fully connected layers. The convolutional layer employs a kernel (a filter window), which is an array of weights, that slides, or convolves, across the input image and multiplies its values to the image’s pixels. The result of the multiplication is an array of numbers, referred to as an activation map or feature map. The pooling layer is used to down-sample the feature map, that is, reduce the spatial size. The down-sampling allows dominant features to be extracted while reducing the computational requirement for data processing. Our CNNs employ the Rectified Linear Units activation function to model neuron’s output. The output of the last convolutional layer is flattened into a single vector and passed through the fully connected layers for classification with a probability of the input belonging to each class.^7^

**Figure 2.**
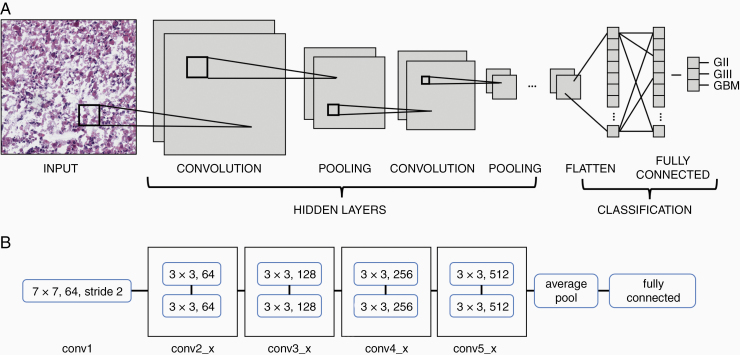
Architectures of CNN and ResNet18 models. (A) Schematic showing the typical architecture of a CNN which involves the input being passed through multiple hidden layers, including convolutional and pooling layers. The weights from the hidden layers are flattened into a single vector and passed through the fully connected layer for classification. (B) Schematic showing the architecture of the ResNet18 model. The first convolutional layer (conv1) with 64 kernels sized 7 × 7 sliding with stride 2. Subsequent convolutional layers are divided into 4 blocks with 2 layers each. The layers employ kernels of size 3 × 3, with layers in the second block (conv2_x) having 64 kernels each, the third (conv3_x) having 128 per layer, the fourth (conv4_x) having 256 per layer, and the last (conv5_x) having 512 per layer. Weights from the last convolutional layer are average-pooled and passed through the fully connected layer for classification.

There are numerous CNN architectures with different depths and varying validation errors. In order to extract more features from the data, there is an inclination to increase the depth (ie, the number of layers) of the model. However, the problems with increasing depth include the vanishing gradient.^11^ Residual neural networks (ResNets) are able to tackle these issues by having layers split into residual blocks and allowing the skipping of layers.^12^ The skipped connection of residual learning allows the ability to train deeper networks without compromising accuracy. Compared to other CNN architectures, ResNets are able to achieve the most depth and best accuracy.^11^ In this regard, the model developed herein follows ResNet18 architecture. The ResNet18 architecture consists of 18 layers, divided into 5 convolutional blocks, an average pool layer, and a fully connected layer^12^ (Figure 2).

### Transfer Learning and Training

In cases where there is limited data, as in the case of our current study, transfer learning may be adopted.^13^ By transferring knowledge learned from one task to learning in a different but related task the learning process is improved.^14^

Our model was trained via transfer learning with layers initialized with weights from the ResNet18 pretrained model using backpropagation. The pretrained ResNet18 has been trained using the ImageNet project.^15^ We further trained the initialized model with our dataset to gain higher level features pertaining to histopathological images of gliomas. The current study utilized the cross-entropy loss function and the stochastic gradient descent (SGD) optimizer. Parallelization was employed in order to train models utilizing multiple graphic processing units and reduce training time. 

The WSIs were split by cases into training (807 cases), validation (201 cases), and evaluation (115 cases) datasets. After data preprocessing, tiles were selected randomly from the training, validation, and evaluation sets, giving a total data split of 60/20/20% training/validation/evaluation. Models were trained in randomly selected batches, where an epoch is one complete feed of the entire training dataset to the model. In order to avoid overfitting, the model training was cut off at the point of validation loss inflection. The weights for the final trained model were taken from the epoch with the highest validation accuracy prior to cutoff.

### Evaluation

The resulting model performance was evaluated on a tile basis using the hold-out evaluation dataset of 115 TCGA patients not used during model training and validation (ie, the model has not seen any images from this dataset). Confusion matrices were calculated. Receiver operating characteristic (ROC) curves were plotted and the areas under the curves (AUROCs) calculated.

For the modular 2-class models, the abovementioned metrics were calculated for the whole classifier. For 3-class classifiers, the metrics were calculated for each class, and the macro-average of all classes for accuracy (ACCM), ROC curves and AUROCs were computed. The macro-average ROC curves were plotted using the macro-average true positive rates (TPRM) and false positive rates (FPRM) at various thresholds.^16^ Metrics were compared between groups using a standard t-test.

### Data Augmentation

Deep neural networks require a large amount of data in order to effectively train the model.^17^ However, where such an amount is unavailable, the dataset may be artificially inflated to increase in size using data augmentation.^18^ In the context of CNNs, augmentation involves transformations to the original images, creating new images for training. Transformations may be geometric, which alter the position and orientation of the image (eg, flip, crop, rescale, and rotation), or photometric, which alter the colors of the image (eg, saturation, contrast, brightness, and hue).^19^

Geometric transformations are the standard in training many CNNs as they provide the most improvement in performance.^19^ For histopathology images, there are considerable photometric variations between samples. This is due to differences in factors affecting stain binding, microscopes, and scanners.^20^ Therefore, we investigated applying both geometrics and photometric transformations in augmentation. Geometric transformations applied were random horizontal flip, random affine (shear factor of 10, scale range of 0.8–1.2). Photometric transformations applied were random color jitter (saturation factor range of 1–2, contrast factor range of 1–2, where 1 is the factor of the original image). 

## Results

### Data Augmentation and Training Parameter Adjustments

**Figure 3.**
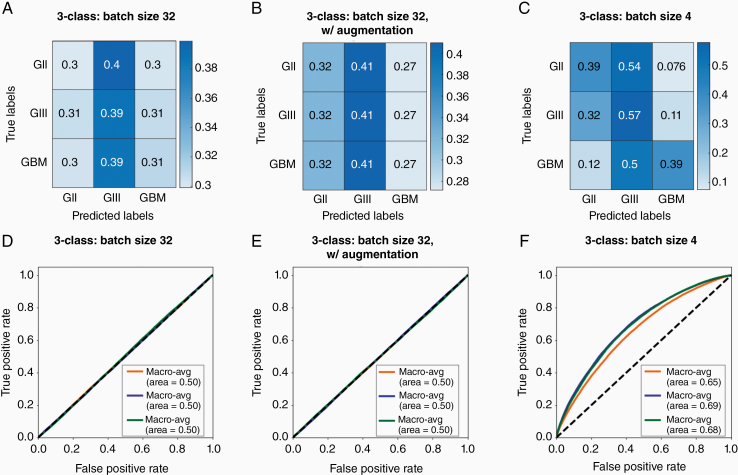
The effects of different augmentation and batch sizing strategies on 3-class classifier model performance. Confusion matrices for 3-class models (A) with geometric but no photometric augmentation and a batch size of 32, (B) with geometric and photometric augmentation and a batch size of 32, and (C) with geometric but no photometric augmentation and batch size of 4. Values are a fraction of the total count for the respective true label (ie, each value is a proportion of the total of its row). Macro-average ROC curves for 3-class models (D) with geometric but no photometric augmentation and a batch size of 32, (E) with geometric and photometric augmentation and a batch size of 32, and (F) with geometric but no photometric augmentation and a batch size of 4. Each line represents the macro-average ROC of all classes for each model run with the same augmentation strategy and hyperparameters, and the area under each macro-average ROC curve is calculated.

The performance of our model differentiating between 3 classes did not improve with the addition of photometric augmentation (mean macro-average accuracy = 56%, mean macro-average AUROC = 0.50) compared to geometric augmentation only (mean macro-average accuracy = 55%, *P* = .751; mean macro-average AUROC = 0.50, *P* = .0745; Figure 3A, B, D , and E) . Moreover, model training became less effective with the addition of photometric augmentation (mean validation accuracy = 35%) compared to geometric augmentations only (mean validation accuracy = 68%, *P* < .01). Hence, photometric augmentations were not used in further development. 

In order to improve model learning and performance, the batch size was adjusted. The model learns the data in batches of randomly selected, unrepeated images from the dataset. An epoch is complete when the entire dataset has been learnt once, and the model weights are updated following each epoch. We compared model and training performance of batch sizes 4 and 32. Batch size of 32 yielded a mean macro-average accuracy of 55% and mean macro-average AUROC of 0.50 at evaluation, while the batch size of 4 yielded improved training performance with a mean macro-average accuracy of 63% (*P* < .001) and mean macro-average AUROC of 0.67 (*P* < .001) at evaluation across models (Figure 3C and F).

### Two-Class vs 3-Class Classification

Previously, Ertosun and Rubin^21^ developed a modular approach to grading brain tumors from histopathology images. This involves the use of 2 separate CNN models, one to classify GBM versus non-GBM and another to classify grade II versus grade III. If the classification obtained from the first model is non-GBM, the input will be passed through the second model to classify the specific grade. 

**Figure 4.**
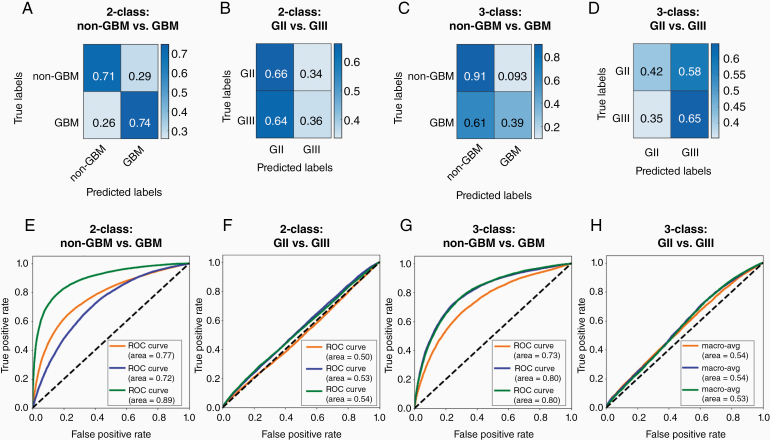
Confusion matrices and receiver operating characteristic (ROC) curves for 2-class and 3-class classifying models distinguishing different classes. Confusion matrices for 2-class models (A) distinguishing between non-glioblastoma (GBM), which includes grades II and III, and GBM and (B) distinguishing between grades II and III. Three-class models were trained to distinguish between all 3 grades. Confusion matrices for 3-class models for classification (C) between grades II and III combined (non-GBM) and GBM and (D) between grades II and III. Values are a fraction of the total count for the respective true label (ie, each value is a proportion of the total of its row). Confusion matrices are used to calculate accuracies. ROC curves are plotted for 2-class classifying models (E) distinguishing between non-GBM and GBM and (F) distinguishing between grades II and III. Within 3-class models, ROC curves are plotted for classification (G) between grades II and III combined and GBM and (H) between grades II and III. Each type of model (ie, two 2-class models and a single 3-class model) was trained 3 separate times, with the same training datasets, augmentation strategy, and hyperparameters. Each ROC curve summarizes a model trained and the area under each ROC curve is calculated.

In our current study, we investigated 2 classification approaches, first a modular approach as proposed by Ertosun and Rubin and second a single 3-class model classifying between 3 grades. Our models distinguishing between GBM and non-GBM achieved a mean accuracy of 72% and mean AUROC of 0.79 at evaluation (Figure 4A and E). The models distinguishing between grade II and grade III obtained a mean accuracy of 51% and mean AUROC of 0.52 at evaluation (Figure 4B and F). Three-class CNN models distinguishing between grade II, grade III, and GBM were trained. For the classification between grades II and III combined (non-GBM) and GBM, the 3-class models obtained a mean accuracy of 73% and mean AUROC of 0.78 (Figure 4C and G). Performance in classifying GBM from other classes is not significantly different between the modular 2-class and 3-class models in terms of accuracy (*P* = .84) nor AUROC (*P* = .53). For the classification of grade II and grade III, the 3-class models obtained mean macro-average accuracy of 53% and mean AUROC of 0.53 between the 2 classes at evaluation (Figure 4D and H). Performance in classifying grades II and III is not significantly different between the 2-class and 3-class models in terms of accuracy (*P* = .10) nor AUROC (*P* = .44).

### Visualization of Outputs and Explanation of CNN

There exists heterogeneity within a tumor in terms of genetic, molecular, and cellular characteristics.^3,22^ Hence, with regard to histopathology, there may be various cellular and morphological characteristics on a single WSI pertaining to different tumor grades, as well as noncancerous tissues. Rather than attempting to calculate an aggregate prediction for the WSI based on the tiles as defined by arbitrary thresholds, it may be preferable to provide localized predictions on a tile basis in order to convey the heterogeneity to clinicians and pathologists.

**Figure 5.**
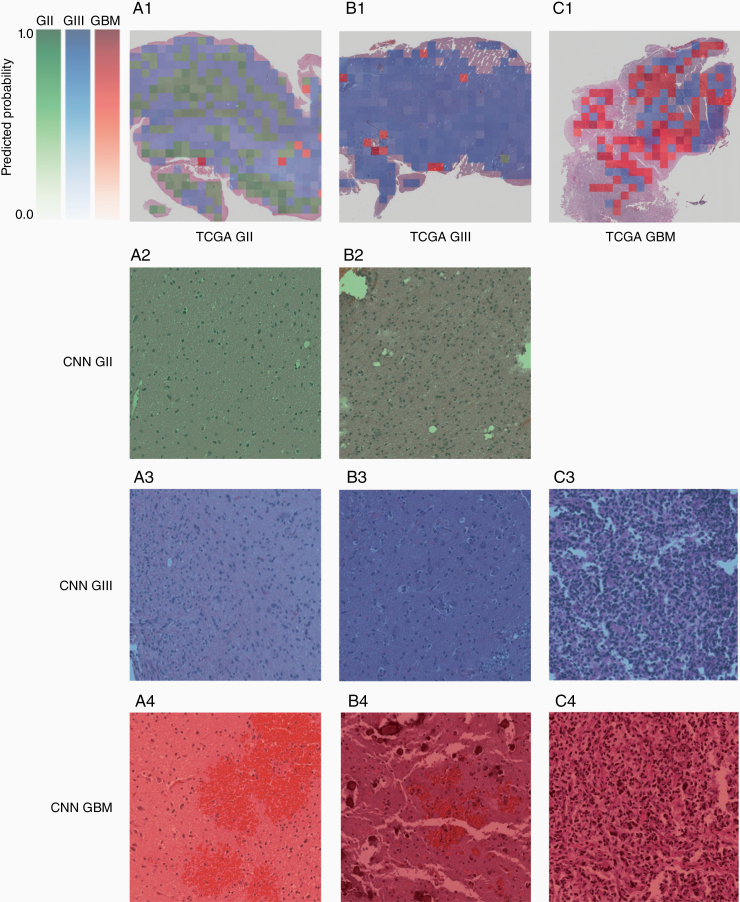
Examples of model output visualization. Whole-slide images (WSIs) are divided into tiles of size 1024 × 1024 at 20× magnification, and tiles with more than 90% tissue are used as inputs for the model. A single voxel is ≈0.501 μm, and an individual tile is ≈513 × 513 μm. Outputs are obtained from the model on a tile basis in terms of scores for each class. The class with the highest score for each tile is chosen for visualization. Each class corresponds with a monochromatic color map, with green corresponding to grade II, blue to grade II, and red to GBM. The intensity of the color is logarithmically proportional to the output score so that higher scores are considerably more intensely visualized relative to lower scores. The scores reflect the model’s confidence in a particular prediction. Examples of (A1) a grade II WSI, (B1) a grade III WSI, and (C1) a GBM WSI with visualized model outputs. For example, individual tiles labeled by CNN as grade II (A2, B2), grade III (A3, B3, C3), and grade IV (A4, B4, C4) are shown for each of the patients (A–C). Considering the grade II patient (column A): a large number of tiles were detected as grade II by the CNN (continued on page 9) Figure 5. (continued from page 8) and indicated regions which showed normal or only a few neoplastic cells (A2); some tiles indicated the possibility of grade III regions which appeared to show higher cellularity (A3), although the confidence of the CNN was in this case low (pale blue); other tiles indicated the possibility of a GBM albeit again in a pale red indicating low confidence and showed regions of hemorrhage (A4). Considering the grade III patient (column B):3 tiles were indicated as grade II and while these regions showed insufficient pathology and cellularity for a grade III diagnosis, it is unclear why tile B2 is labeled as grade II while tile B3 is labeled as grade III. This highlights the importance of, in parallel, developing methods that indicate the regions of the tile that were considered most important by the CNN in making a diagnosis as discussed in Section *Visualization of Output and Tumor Heterogeneity*. Furthermore, within patient B, regions detected as GBM showed regions of hemorrhage (B4) similar to A4. Considering the GBM patient, no tiles were detected as grade II; regions detected as grade III were less vascular and slightly less polymorphic (C3) as compared to regions detected as GBM (C4).

An algorithm was written to visualize the output of the model. A WSI provided is divided into tiles and each tile satisfying the abovementioned criteria is subsequently passed through the model to obtain predicted probabilities of belonging to each class. The class with the highest predicted probability will be visualized. Each class is associated with a monochrome color map ranging from light to dark: green represents grade II, blue represents grade III, and red represents GBM. The probability translates logarithmically to the color map so that lower probabilities (<50%) are displayed as very light in color and rapidly increases in intensity at higher probabilities (≥50%). The color determined by the class and probability is overlaid on the tile. The result is a heatmap corresponding to the class of with the highest probability and such probability overlaid over the original WSI (Figure 5). A selection of tiles was also reviewed by a consultant histopathologist (C.L.-S.) and shown in Figure 5.

## Discussion

We investigated the use of deep learning in classifying brain tumor histopathological images for grading. Several CNN models were trained with varying strategies, including data augmentations, batch size adjustments, and 2-class versus 3-class classifiers. A methodology for visualizing the output in order to highlight heterogeneity was developed to aid clinicians in diagnosis. 

### Data Augmentation Strategies

The effectiveness of learning and ultimately the performance of a machine learning model are dependent on the characteristics of the dataset provided and the architecture of the model with its hyperparameters.^23–25^ For this study, we looked to optimize our training dataset through data augmentation and adjusting model hyperparameters, specifically batch size, to maximize learning and performance. 

Data from the TCGA are obtained from multiple centers, hence there are variabilities in protocols for tissue processing, staining, microscopy imaging, and digitalization, which introduce a myriad of variance in image properties.^10^ Therefore, we looked to augment the dataset with photometric transformations to imitate the variations in saturation, contrast, and brightness. However, our photometric augmentation compromised learning, contrary to what was expected. Although in theory data augmentation has been thought to improve learning, its effectiveness in providing meaningful data has also been discussed previously. The alterations and transformations offered by the traditional methods of data augmentation are relatively minimal and do not offer new, indistinguishable information from the original data which could significantly improve learning and subsequently predictive capabilities.^26^

Depending on the nature of the dataset and the type of augmentation, photometric transformations have been shown to reduce model performance.^15,27,28^ In the current study, the transformations performed on the images were randomized. We provided ranges for the transformation factors, and the algorithm computed a random value within those ranges for the different transformations, namely, saturation, contrast, and brightness. There may have been transformations to the images where the color properties no longer fall within the normal ranges of the unmodified data. Such modification may also remove important features and class-dependent color information, thus producing data not reflective of the label. Hence the augmented data increase the complexity within the training dataset, providing unrealistic and out-of-context inputs to the model, impairing the ability of the model to effectively learn.^29^

One promising data augmentation technique is generative adversarial networks (GANs). GANs in themselves are a type of machine learning network which aim to generate, hence generative, new, synthetic data that are indistinguishable from the original training dataset.^30^ This allows the dataset to be artificially inflated with greater modifications and more variability in features compared to classical data augmentation and potentially enrich the training dataset further. Combined with classical data augmentation, the strategy has the potential to inflate the data to a considerable extent (up to 100 times).^30^ GANs have been shown to be useful in augmenting datasets and improve machine learning performance in the context of medical imaging, especially when combined with classical data augmentation techniques.^31,32^ Hence, in future work, GANs are an avenue of exploration for further improving model training and performance.

### Hyperparameter Adjustments

Algorithm hyperparameters are factors influencing model accuracy and loss, and hence are often fine-tuned to optimize performance, which can be in itself a complex and difficult task.^33^ One important hyperparameter is training batch size. There is no particular generalized formula or rule-of-thumb in determining the batch size for optimal learning. Batch size choice is dependent on the objective of training (eg, increase training speed and increase generalizability), thus there are conflicting conclusions on whether increasing or decreasing batch size would result in better performance. Larger batch size has been evidenced to produce higher classification accuracy,^25^ aside from increasing training speed.^34^ However, in tasks where generalizability is a major consideration, larger batch sizes have been shown to degrade model quality, particularly with the SGD optimization method used herein.^35^ Smaller batch sizes also prevent overfitting as the resulting gradient noise acts as a good regularizer, avoiding sharp minima and thus leading to better generalization.^35,36^ For histopathology particularly, and medical imaging as a whole, generalization is an important consideration due to the great interindividual and intertumor heterogeneity.^3,22^ This is reflected in our current study, where a smaller batch size of 4 produced superior model learning and performance compared to a batch size of 32.

### Two-Class vs 3-Class Models

We tested 2 methods for classifying tumor histopathology images into multiple grades: (1) a modular approach involving two 2-class models, first between GBM and non-GBM and then between grades II and III within the non-GBM group as previously proposed by Ertosun and Rubin and (2) a single 3-class model distinguishing between grades II, III, and GBM. 

It is generally evidenced that 2-class models are more effective at learning compared to one classifying multiple classes simultaneously as they are less complex.^37–39^ It is also suggested that multiclass systems demand more specific hyperparameter tuning and optimization.^40^ On the other hand, several 2-class classifiers collectively require more training time as well as have slower classification speed, due to the input being passed through various layers of classification, as compared to a single multiclass classifier.^40^

It was observed in this study that multiple 2-class classifiers did not significantly outperform a single 3-class classifier. Furthermore, the two 2-class models collectively took approximately twice as long to train per epoch compared to a single 3-class model in our case. In this respect, for the task presented, there appeared to be no advantage of a 2-class system over a 3-class system. There is also evidence in the literature of multiclass classifiers outperforming multiple 2-class classifiers.^40^ Results from our study suggest that the use of a 3-class system is more appropriate for grading histopathological images. This is particularly important as the task in clinical practice would require a quick and responsive system, especially if the technique is to be extended to provide real-time feedback with on-line microscopy. 

Previously Ertosun and Rubin attempted the same classification task for brain tumor histopathological images from the TCGA database using 2-class classifiers and achieved 96% accuracy in distinguishing GBM from grades II and III combined and 71% in classifying between grade II and grade III.^21^ The model in the current study did not achieve accuracies as high. While their approach involved extracting the nuclei of cells from histopathology images and only using this feature for training, our approach utilizes the raw images with minimal preprocessing. This includes and utilizes more features in training aside from the nuclei, for example, cellular morphology and vascularization of the tumor. However, in attempting to integrate as many features as possible, the complexity of the data was considerably increased, rendering learning more complex. Another important aspect of our approach is the use of transfer learning with initialized weights from the ResNet18 model, whereas Ertosun and Rubin trained their CNNs from randomly initialized weights. This may partially account for the discrepancy between our findings as discussed in the next section. 

The models performed better in distinguishing GBM from other grades. This is to be expected as characteristics of GBM are more distinctive than those pertaining to other grades.^21^ On the other hand, classification between grades II and III is more clinically challenging. This is reflected in the poor performance observed in both of our strategies (close to random chance), as well as the relatively inferior performance to GBM classification observed by Ertosun and Rubin.^21^

Nevertheless, in the current study neither strategies, 2-class nor 3-class, have produced models with optimal performance. This can be mainly attributed to the training dataset, where the labeling did not permit effective learning. A WSI may contain normal tissue aside from tumor tissue, which were included in the training dataset and labeled as tumors. Furthermore, there is heterogeneity in a WSI which may contain tissues with different histopathological features pertaining to different grades. In practice, a pathologist would be able to identify the heterogeneity in characteristics and assign a grade based on the present features associated with the highest grade.^4^ However, this heterogeneity had not been accounted for and the entire WSI and its constituent tiles are labeled with the patient’s diagnosed grade. Hence this imprecision in the labeling of the training data impaired the effectiveness of learning and subsequently performance of the models. This could potentially be addressed in the future by using weakly supervised learning systems as suggested in Ref. ^41^.

### ResNet18 and Transfer Training

Through transfer learning, weights are initialized from a well-established CNN to exploit the features learned rather than random initialization. Here we utilized the ImageNet weights for basic features such as edge detection and contrast, however transferring from histological datasets may offer more relevant features.^42^ While this is usually advantageous, it may still pose a challenge in finding the optimum model for a task highly divergent from the dataset of the transferred model. A recurrent problem in developing neural networks generally, and transfer learning particularly, is the local minima problem.^43^

Initialization with specific weights, as with the case of transfer learning, puts the model at a specific point on the loss landscape. As such training of multiple models in the same manner is likely to obtain similar results as the models ultimately reach the same local minima. This restricts the ability of the algorithm to potentially reach the global minimum, or other more optimal local minima, which would otherwise be possible with the random initialization, but at the expense of training time as well as the risk of overfitting. Nevertheless, random initialization is a straight-forward and popular method to avoid the local minima problem.^44^ Other strategies, such as simultaneous learning or hidden nodes, have been proposed which could be explored in the future.^45,46^

### Visualization of Output and Tumor Heterogeneity

One approach for utilizing digital pathology deep learning models is to obtain a single classification output for each WSI input to the CNN. Due to the large dimension of WSI, the slide is sectioned into tiles and the outputs were given on a tile basis. In order to obtain a single output per WSI, the tile outputs would need to be aggregated. Several options were considered, including average voting, that is, taking the average score of all tiles for each class and the final output is the class with the highest average score, and maximum voting, that is, taking counts of the class with the highest score from each tile and the final output is the class with the highest counts. 

However, neither of these strategies are reflective of the decision-making process in practice. Due to the intratumor heterogeneity, where characteristics of multiple grades are exhibited in a sample, the pathologists are inclined to assign the highest possible grade with features present.^47^ In which case, there are multiple thresholds which need to be defined in order to implement the abovementioned strategies with confidence, for example, the proportion of the higher class that needs to be present and the minimum average score of the higher class in order to overrule the lower class’ majority. It is challenging to define such thresholds in a clinically relevant manner due to the subjective nature of the task. Grading guidelines are imprecise and thus individual histopathologist and clinician would have their own personal criteria and an interobserver variation in grading glioma exists.^4^

Hence, rather than attempting to define arbitrary thresholds, which further magnifies the problem of subjectivity, we aimed to provide a tool to aid in the clinical decision-making process rather than attempt to replace it with machine learning. Accordingly, the outputs are visualized on a tile basis, which highlights the different classes predicted and thus heterogeneity within a sample. This helps to guide the reading by bringing attention to localized features. This method (Figure 5) was developed with a consultant histopathologist (C.L.-S.) with the vision of creating a tool that is able to identify the most aggressive regions of a WSI. Furthermore, we discussed the use of machine learning tools in the clinic with a brain tumor patient and public involvement group and while there is support for using computers to improve the diagnostic processes, patients are weary of having a computer making a final diagnosis. The method we describe would potentially speed up the diagnostic process and reduce interobserver variability, while also addressing patient concerns. 

Nevertheless, before this technique can be implemented in the clinic, the accuracy of the network needs to be improved by addressing the various points raised within this study. Subsequently, the method may be evaluated and validated. One such way is pathologists’ analysis of selected tiles with high confidence to evaluate any biases, as well as identifying any correspondence to key diagnostic features or any recurrent features utilized by the model that may not be part of the clinical criteria. We have demonstrated this in Figure 5, showing how review by pathologists of a selection of individual tile classification by CNN can give feedback to the developers to further improve the system. Through review we noted that the machine learning network tended to misclassify regions of hemorrhage as GBM. Due to the presence of abundant and often structurally abnormal blood vessels, GBMs tend to have increased regions of hemorrhage compared to lower grade tumors, and this could be resulting in the machine learning network learning to classify these nondiagnostic regions of a tumor as GBM. This result indicates the importance that the machine learning network gave to the vasculature and may also explain the difference in accuracy seen between our study and the study by Ertosun and Rubin^21^ which focused on nuclei. This result could guide the further development of the network through additional training that ensures that nondiagnostic areas of hemorrhage are not used within the classification and observe whether this improves the classification accuracy. That said, we are currently making an assumption that the machine learning network focused on these regions of hemorrhage which are evident within the misclassified tiles. Visualization of the network decisions may involve within tile assessment through other techniques such as Grad-CAM^48^ to highlight areas within the image deemed more important for prediction by the CNN. This could then confirm that, for example, the hemorrhage regions were indeed considered the most critical within that individual tile for classification. The technique may therefore provide further guidance in building a robust and reliable network that is able to conduct grade assessment more specifically within each of the tiles.^48^

### Quality and Scope of Dataset

One important aspect to the success of a machine learning model is the quality of the input data. There are 2 main limitations of the TCGA dataset with respect to the analysis carried out, which are likely to have affected the performance of our methods.

First is the tissue preparation method used in the dataset. Most of TCGA’s glioma samples (68% of non-GBM and 75% of GBM samples) were fresh frozen, which often results in a loss of tissue morphology due to freezing artifacts.^49^ Model performance could be improved by utilizing samples prepared from a technique which allows for better preservations of cellular and architectural morphology, for example, formalin-fixed paraffin-embedded.^50^

Second, the TCGA dataset was put together prior to the WHO 2016 classification of brain tumors^2^ and therefore lacks additional molecular information. TCGA’s dataset reports IDH and 1p/19q mutation status for roughly half of the cases.^51,52^ Previously, improved performance has been shown when taking into consideration IDH and 1p/19q mutation status in predicting prognosis.^53^ Nevertheless, the model should not rely on nor weigh heavily on the molecular data as an input as these are not always available.^54^ In our study, we put an emphasis on developing explainable machine learning systems to grade brain tumors based on tumor morphology. However, genetic profiles would be important in future studies and should, at least initially, be considered separately from morphology in order to build and assess explainable machine learning models that integrate both computational and pathological expertise. A number of studies looked into the computational prediction of mutational status, for example, IDH,^55^ 1p/19q,^56^ and TP53,^57^ using histopathological images. This may be useful where the information is required by the histologist for diagnosis but the test has failed or could not be performed and could be incorporated into our approach in the future.

Future studies would greatly benefit from the availability of a highly curated dataset with a wider scope of molecular markers and acquired using techniques that preserve tissue morphology. Furthermore, using a well-curated dataset can ensure that the primary features used in grading tumors clinically (eg, cellularity, mitotic activity, vascular proliferation, and necrosis) are visible at the magnification in which the images are acquired and will allow for use of key diagnostic criteria to improve model performance. 

## Conclusion and Future Perspective

Deep learning, and specifically CNNs, may improve digital pathology analysis of brain tumors. We developed a methodology to visualize a predictive tumor grading model on histopathology images to aid and guide the clinicians by highlighting features and underlining the heterogeneity in predictions. However, the accuracy of the trained network was low, particularly in differentiating between WHO grade II and III gliomas. Many models have been developed to grade gliomas using machine learning architectures other than CNNs and have shown high performance.^58,59^ Thus, exploration outside of CNN may be of interest for further investigations.

In the context of machine learning methods, in order to improve classification on standard H&E stains, there is a need for a well-curated dataset that includes molecular characteristics and that employs preparation techniques which preserve tissue morphology. Furthermore, methodology development is required before such tools can be implemented clinically, particularly related to the issue of tile versus WSI labels, data augmentation, and model optimization techniques. In our work, a selection of tiles was reviewed by a consultant histopathologist for evaluation of the machine learning tool. Future work would need to include expert evaluation of the tiles categorized by the machine learning network as critical, and these would need to be evaluated with reference to whether they correspond to key features of increased malignancy. The development of the CNNs needs to take place in conjunction with the histopathologist tile review to continually update and optimize the methods developed. This would lead to further fine-tuning of the networks developed, improving system reliability. Finally, while machine learning tools have the potential of aiding clinicians, these need to be developed in a strong collaboration between end-users, as well as clinical and computing scientists, and a strong involvement from patient and carer groups.
